# Squamous cell carcinoma arising from Warthin’s tumor in the parotid gland

**DOI:** 10.1259/bjrcr.20190032

**Published:** 2019-11-15

**Authors:** Ji-Eun Kim, Tae Gyu Kim

**Affiliations:** 1 Department of Radiology, Inje University Sanggye Paik Hospital, Seoul, Korea

## Abstract

The malignant transformation of Warthin’s tumor in the parotid gland is extremely rare. In addition, among the various types of the malignant change of Warthin’s tumor, squamous cell carcinoma is the rarest one. The pathogenesis of this rare disease entity is uncertain till now. Also the most effective treatment and long-term prognosis are not yet clear. Up to present, there are a few reports that only described the pathologic and clinical features of this tumor. However, there is no report about the imaging findings of this rare tumor. We report the imaging features in a case of squamous cell carcinoma arising from Warthin’s tumor in the parotid gland.

## Introduction

Warthin’s tumor is the second most common benign neoplasm of the salivary gland and accounts for 5–10% of all parotid tumors.^1^ It is usually an asymptomatic and slow-growing mass. Meanwhile, the conversion of Warthin’s tumor into malignant carcinoma is extremely uncommon (0.3%).^2^ If malignant conversion or inflammation occurs in it, abrupt increase of its size would be noted.^3^

Histologically, Warthin’s tumor is composed of epithelial and lymphoid elements. And as regards malignant change of Warthin’s tumor, it is relatively common that the lymphoid stroma evolves into malignant lymphoma. Meanwhile, epithelial malignancy is highly uncommon.^2^ Adenocarcinoma, mucoepidermoid carcinoma, and squamous carcinoma can develop from the epithelial element.^1,4^ And among them, malignant transformation of the tumor to squamous cell carcinoma is the rarest one. Till date, only eight cases of squamous cell carcinoma arising from Warthin’s tumor have been reported.^1,2,5–7^ We report the imaging features of squamous cell carcinoma arising from Warthin’s tumor.

## Case presentation

**Figure 1.**
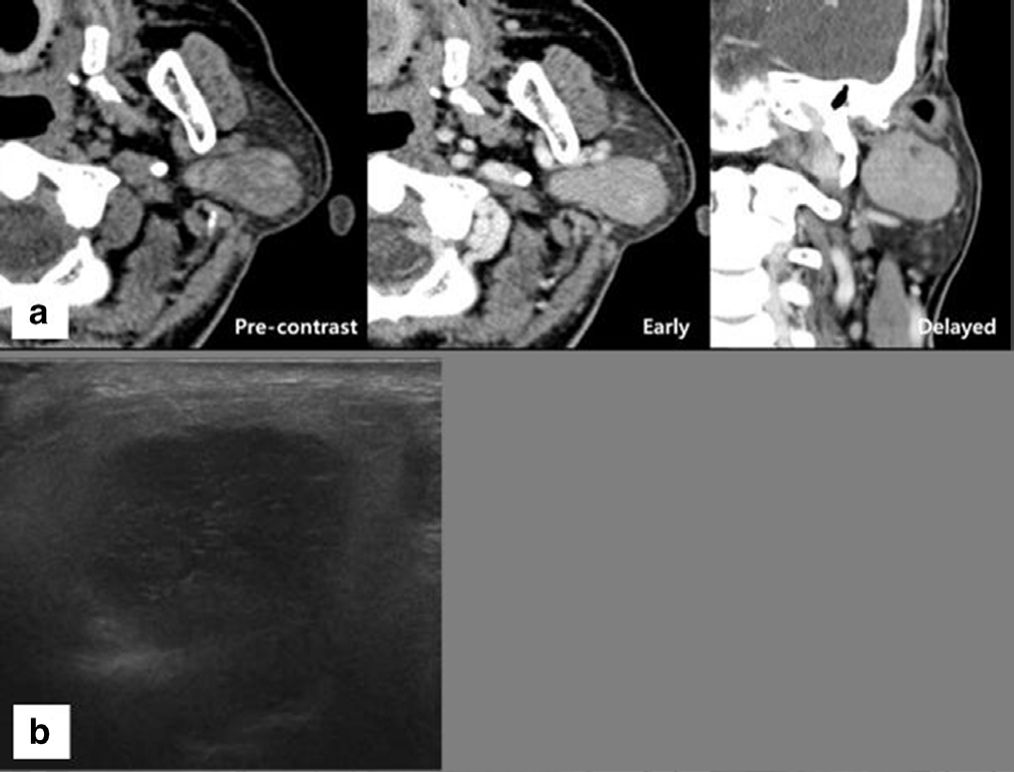
A 68-year-old male with slow-growing mass in the left preauricular area. (a) A well-marginated heterogeneously attenuated (32~78 HU) soft tissue mass measuring about 3 cm shows dumbbell shape involving both superficial and deep lobes of the left parotid gland on axial precontrast CT scan. After 60 s from the injection of contrast media, the tumor shows dense enhancement (100 HU) on axial CT scan. After 120 s from the injection of contrast media, no definite contrast-washout nor gradual enhancement of the mass (100 HU) is noted on coronal CT scan. (b) Sonogram shows a well-defined heterogeneous hypoechoic mass in the left parotid gland. HU, Hounsfiled unit.

**Figure 2.**
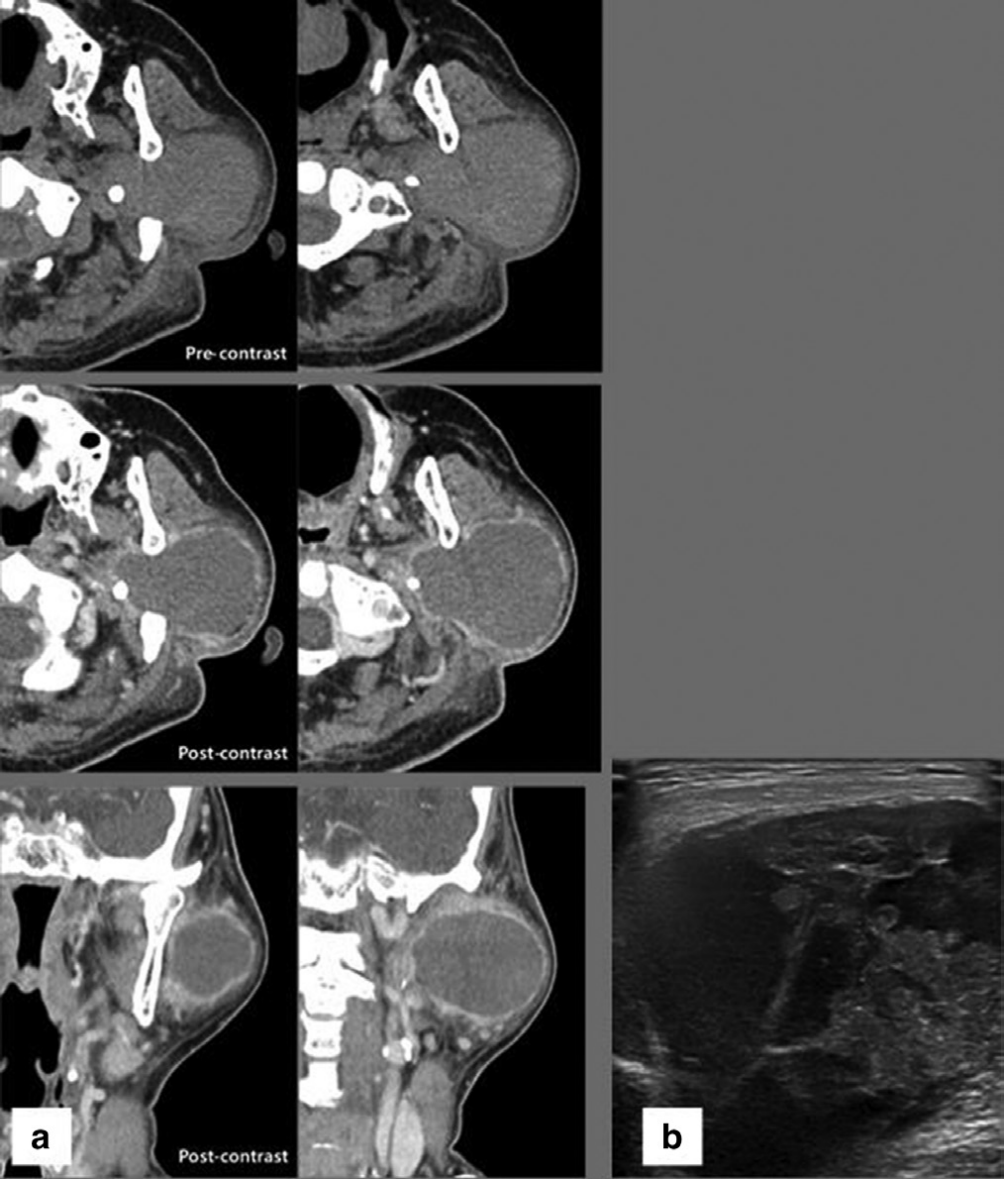
The same patient as Figure 1 with rapidly growing tender mass in the left preauricular and submandibular areas, after 8 years from initial imaging studies. (a) Pre-contrast axial CT scan reveals approximately 7 cm-sized homogeneously attenuated mass with perilesional fat infiltrations in the left parotid gland. The attenuation of its internal low density portion is measured as 47 HU. Contrast enhanced axial and coronal images reveal non-enhancing internal low density portion and peripheral thick enhancing rim of the mass. There is no definite enhancing nodular portion in the mass. (b) Sonogram shows large solid and cystic mass in both superficial and deep lobes of the left parotid gland. HU, Hounsfiled unit.

**Figure 3.**
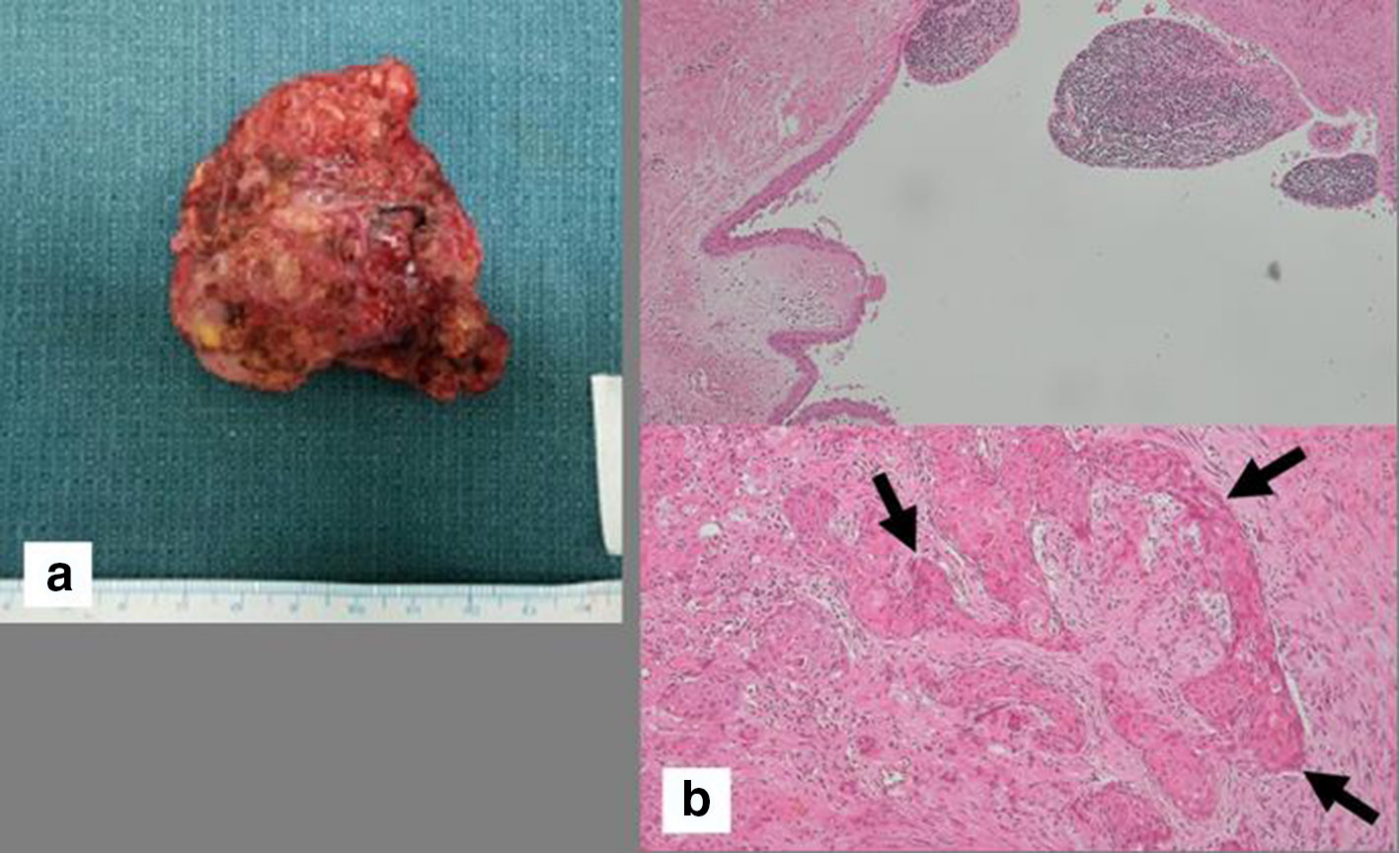
Pathologic findings of the mass in the left parotid gland. (a) Gross specimen of resected left parotid gland shows a 6 cm-sized firm mass with cystic change. (b) Microscopic specimens of the left parotid tumor. The upper panel shows squamous cell-lined cyst (H&E staining, x 40). And the lower panel shows Warthin’s tumor with infiltration of stroma by malignant squamous cells (arrows) (H&E staining, x 100).

A 68-year-old male presented with a slow-growing painless mass in his left preauricular area, detected 4 months ago. On physical examination, a non-tender lump measuring 3 cm in size was palpated in the left parotid region and partly fixed to underlying structures. Clinically, there was no evidence of facial nerve involvement nor regional lymphadenopathy. Laboratory tests showed the complete blood count and inflammation indexes within normal limits. CT revealed approximately 3 cm sized well-marginated heterogeneously attenuated soft tissue mass in the left parotid gland, involving both superficial and deep lobes, making the dumbbell shape. Contrast-enhanced images showed dense enhancement. However, on the delayed image, no definite contrast-washout nor gradual enhancement of the mass was noted (Figure 1a). Sonogram showed a heterogeneous hypoechoic mass in the left parotid gland (Figure 1b). And ultrasound-guided fine-needle aspiration cytology (FNAC) revealed organizing abscess, which is not corresponding with the characteristic of radiologically dense enhancement of the mass, suggesting unsatisfactory cytologic result. Despite the unsatisfactory cytologic result, the patient was advised to undergo left superficial parotidectomy for the possibility of malignancy, but he refused. After then, the size of the mass continued to grow. After 8 years, the patient presented with rapid swelling and tenderness of the left preauricular and submandibular areas. CT revealed the left parotid mass had grown up to approximately 7 cm, revealing internal non-enhancing low density portion and peripheral enhancing rim with perilesional fat infiltrations. The attenuation of its low density portion was measured as 47 HU. There was no definite enhancing nodular portion (Figure 2a). Sonogram showed large solid and cystic mass in both superficial and deep lobes of the left parotid gland (Figure 2b). The second ultrasound-guided FNAC was performed and the possibility of cystic metastasis, such as very well differentiated squamous cell carcinoma was suggested. Under general anesthesia, the patient underwent enucleation of the parotid tumor. Grossly, his left parotid gland was mostly replaced with cystic mass, measuring about 6 cm (Figure 3a). During the procedure, the cystic mass was ruptured and discharge of necrotic materials was noted. Histologic microscopy of the left parotid tumor revealed Warthin’s tumor with infiltration of stroma by malignant squamous cells (Figure 3b). The patient was finally diagnosed as squamous cell carcinoma arising from Warthin’s tumor of the left parotid gland. Positron emission tomography-CT (PET-CT) after surgery showed the absence of abnormal uptake, suggesting the left parotid gland was the only primary site.

## Discussion

Squamous cell carcinoma arising from Warthin’s tumor is extremely rare and the etiology of it is not established.^1,2,5,6^ Previously, Damjanov et al^8^ supposed that squamous cell carcinomas arose from a focus of squamous metaplasia. The presence of cytoplasmic keratin in the Warthin’s tumor cells may provide a clue to the case with which these cells undergo squamous metaplasia.^5^ The transition from cylindrical cells to squamous cells may be due to infection or ischemic change in a large tumor. Ischemia was thought to be the most probable etiology for squamous metaplasia.^1^

In addition, it is important to discriminate it from the metastasis of primary squamous cell carcinoma at other sites.^8^ Among the majority of reported cases, in comparison to their uncommon distant metastasis, regional metastatic lymphadenopathy was common.^1,7^ We did not discover any distant nor regional lymph node metastasis in our case.

There are a few reports that described the pathologic and clinical features of this tumor. However, up to present, there is no report about the imaging findings of it. In terms of well-defined parotid tumor, one of CT features of Warthin’s tumor is enhancing pattern, strong enhancement on early phase and decreased enhancement on delayed phase, suggesting “Washout.” On the other hand, most of pleomorphic adenomas show progressive enhancement on CT scan.^9^ However, in the initial CT scan of this case, there was no definite contrast washout nor gradual enhancement on delayed image. Another interesting point is the mass was located in both superficial and deep lobes of parotid gland, making the dumbbell shape, in the initial CT scan. It is one of the characteristics of pleomorphic adenoma, distinct from Warthin’s tumor.^10^ In case of Warthin’s tumor, it tends to be located in the posterior aspect of superficial lobe of parotid gland. Furthermore, most of the malignant carcinomas contain enhancing nodular portions on CT images. But in this case, there is no definite enhancing nodular portion in the mass, except for the peripheral thick enhancing rim.

## Conclusion

The imaging findings of squamous cell carcinoma arising from Warthin’s tumor in this case differ from the typical findings of other malignant carcinomas and benign Warthin’s tumor. Moreover, the most effective treatment and long-term prognosis of this tumor are not yet clear.^11^ In this report, we present a case of squamous cell carcinoma arising from Warthin’s tumor, together with its imaging features. More reports of new cases will be necessary to describe the imaging features of this rare disease entity. Although the definitive diagnosis requires a histologic confirmation after surgery, knowledge of its imaging features before invasive procedures will help the clinician to make appropriate decision.

## Learning points

1. The malignant transformation of Warthin’s tumor in the parotid gland is extremely rare. And among the various types the malignant change of Warthin’s tumor, squamous cell carcinoma is the rarest one.
2. The imaging findings of squamous cell carcinoma arising from Warthin’s tumor in this case are quite different from the typical findings of other malignant carcinomas. In this case, there is no definite enhancing nodular portion in the mass, except for the peripheral thick enhancing rim.
3. Also, the initial imaging finding of this tumor contains characteristics of both of Warthin’s tumor and pleomorphic adenoma.
